# *In Vivo* Pharmacodynamic Study of Cefiderocol, a Novel Parenteral Siderophore Cephalosporin, in Murine Thigh and Lung Infection Models

**DOI:** 10.1128/AAC.02031-18

**Published:** 2019-08-23

**Authors:** Rio Nakamura, Tsukasa Ito-Horiyama, Miki Takemura, Shinsuke Toba, Shuhei Matsumoto, Tatsuya Ikehara, Masakatsu Tsuji, Takafumi Sato, Yoshinori Yamano

**Affiliations:** a Drug Discovery & Disease Research Laboratory, Shionogi & Co., Ltd., Osaka, Japan

**Keywords:** *Acinetobacter baumannii*, CRE, *Pseudomonas aeruginosa*, PK/PD, *Stenotrophomonas maltophilia*, cefiderocol, *in vivo*, lung infection, multidrug resistance, thigh infection

## Abstract

The pharmacokinetic (PK) and pharmacodynamic (PD) parameters which correlated with the *in vivo* efficacy of cefiderocol were evaluated using neutropenic murine thigh and lung infection models in which the infections were caused by a variety of Gram-negative bacilli. The dose fractionation study using the thigh infection model in which the infection was caused by Pseudomonas aeruginosa showed that the cumulative percentage of a 24-h period that the free drug concentration in plasma exceeds the MIC (%*fT*_>MIC_) rather than the free peak level divided by the MIC (*fC*_max_/MIC) and the area under the free concentration-time curve over 24 h divided by the MIC (*f*AUC/MIC) was the PK/PD parameter that best correlated with efficacy. The study with multiple carbapenem-resistant strains revealed that the %*fT*_>MIC_ determined in iron-depleted cation-adjusted Mueller-Hinton broth (ID-CAMHB) better reflected the *in vivo* efficacy of cefiderocol than the %*fT*_>MIC_ determined in cation-adjusted Mueller-Hinton broth (CAMHB). The mean %*fT*_>MIC_ of cefiderocol required for a 1-log_10_ reduction against 10 strains of Enterobacteriaceae and 3 strains of Pseudomonas aeruginosa in the thigh infection models were 73.3% and 77.2%, respectively. The mean %*fT*_>MIC_ for Enterobacteriaceae, P. aeruginosa, Acinetobacter baumannii, and Stenotrophomonas maltophilia in the lung infection model were 64.4%, 70.3%, 88.1%, and 53.9%, respectively. These results indicate that cefiderocol has potent efficacy against Gram-negative bacilli, including carbapenem-resistant strains, irrespective of the bacterial species, in neutropenic thigh and lung infection models and that the *in vivo* efficacy correlated with the *in vitro* MIC under iron-deficient conditions.

## Abstract (precis)

The pharmacokinetic (PK) and pharmacodynamic (PD) parameters which correlated with the *in vivo* efficacy of cefiderocol were evaluated using neutropenic murine thigh and lung infection models in which the infections were caused by a variety of Gram-negative bacilli.

## INTRODUCTION

Carbapenem antibiotics, such as meropenem, have been used for the treatment of serious health care-associated infections, including complicated urinary tract infection, sepsis, and pneumonia, due to Gram-negative pathogens. However, the increase of carbapenem-resistant (CR) Gram-negative bacteria has become a serious global health care issue. CR Enterobacteriaceae (CRE), CR Pseudomonas aeruginosa (CRPA), and CR Acinetobacter baumannii (CRAB) are listed by WHO as critical pathogens which cause the greatest threat to human health (1). The development of new antibiotics is urgently needed, as these pathogens can cause severe and often deadly infections, such as bloodstream infections and pneumonia (1, 2). Stenotrophomonas maltophilia is also an intrinsically carbapenem-resistant pathogen due to the chromosomally encoded L1 metallo-β-lactamase (MBL) and causes an increasing number of nosocomial infections (3). As S. maltophilia exhibits resistance to a broad array of antibiotics, the clinical therapeutic options are scarce or are limited to trimethoprim-sulfamethoxazole (4).

Recently, new combination products consisting of a β-lactam and a β-lactamase inhibitor, such as ceftazidime-avibactam, ceftolozane-tazobactam, and meropenem-vaborbactam, have become new therapeutic options for infections caused by these CRE. However, none of these antibiotics are active against MBL-producing CRE, carbapenemase-producing CRPA, CRAB, or S. maltophilia.

Cefiderocol (S-649266) is a novel parenteral siderophore cephalosporin with a catechol moiety on the 3-position side chain discovered by Shionogi & Co., Ltd. This unique structural feature enables cefiderocol to bind to ferric iron and be actively transported across the outer membrane of Gram-negative bacteria via ferric iron transporters, which means that cefiderocol activity is enhanced under iron-limited conditions due to the inducible production of iron transporters (5, 6). In addition, cefiderocol is highly stable to both serine- and metallo-type carbapenemases, such as KPC-3, IMP-1, VIM-2, L1, and NDM-1 (7). Due to these unique features, cefiderocol shows potent *in vitro* activity against a variety of Gram-negative pathogens, including CRPA, CRAB, CRE, and S. maltophilia, irrespective of the production of serine- and metallo-type carbapenemases (8–11).

The goals of our studies were (i) to clarify the pharmacokinetic (PK) and pharmacodynamic (PD) parameter which best correlates with *in vivo* efficacy, (ii) to determine appropriate MIC culture conditions for cefiderocol to predict *in vivo* efficacy, and (iii) to determine the magnitude of the PK/PD parameter for bactericidal activity.

## RESULTS

### Susceptibility of test strains to cefiderocol.

The MICs of cefiderocol against 24 test strains, determined using iron-depleted cation-adjusted Mueller-Hinton broth (ID-CAMHB) and cation-adjusted Mueller-Hinton broth (CAMHB), are summarized in Table 1. Since cefiderocol is designed to utilize ferric iron transporters, which are induced under iron-deficient conditions, to efficiently penetrate the bacterial outer membrane, cefiderocol generally showed greater *in vitro* activity under iron-deficient conditions. The degree of the MIC change between ID-CAMHB and CAMHB varied depending on the strain.

**TABLE 1:** MICs of cefiderocol, cefepime, and meropenem against the tested strains

Organism	Type of carbapenemase	Infection model applied in this study	MIC (μg/ml)			
			Cefiderocol		Cefepime	Meropenem
			ID-CAMHB	CAMHB		
E. coli						
ATCC 25922	—^*d*^	Thigh	0.125	0.25	0.125	≤ 0.031
AB	NDM-4	Lung	4	8	>32	32
IR-5	NDM-1	Lung	4	4	>32	>32
K. pneumoniae						
ATCC 13883	—	Thigh	0.25	0.5	0.063	≤ 0.031
1478266	—	Thigh	0.5	1	0.063	0.063
1478677	—	Thigh	0.25	0.25	>32	0.125
VA-357^*a*^	KPC-2	Thigh, lung	2	8	>32	32
VA-361^*a*^	KPC-2	Lung	4	16	>32	16
VA-384^*a*^	KPC-2	Thigh, lung	4	16	>32	>32
VA-391^*a*^	KPC-3	Thigh, lung	4	16	>32	16
6560-MAR^*b*^	NDM-1	Thigh, lung	2	2	>32	32
KI2^*c*^	NDM-1	Thigh, lung	8	32	>32	>32
NCTC 13443	NDM-1^*c*^	Thigh, lung	16	256	>32	>32
P. aeruginosa						
SR27016	—	Thigh	0.25	0.25	1	0.5
ATCC 27853	—	Thigh, lung	0.5	2	2	0.25
SR27001	IMP-1	Thigh, lung	2	32	>32	>32
NCTC 13437	VIM-10	Lung	1	8	>32	>32
A. baumannii						
BEN ST BRI	OXA-24	Lung	0.25	2	>32	>32
1485247	—	Lung	2	32	>32	8
NCTC 13301	OXA-23	Lung	1	32	>32	32
S. maltophilia						
1146824	Not tested	Lung	0.125	0.125	>32	>32
1371071	Not tested	Lung	0.125	0.125	>32	>32
1392567	Not tested	Lung	0.25	0.25	>32	>32
1444463	Not tested	Lung	0.25	0.125	>32	>32

a ST258.

b ST15.

c ST14.

d —, not detected.

### Pharmacokinetics of cefiderocol in neutropenic murine infection models.

The PK parameters of the total concentrations of cefiderocol and cefepime in the plasma of the mice used for the murine thigh and lung infection models are shown in Table 2. When cefiderocol was administered subcutaneously at doses of 4, 40, and 400 mg in the thigh infection model, the maximum plasma concentration (*C*_max_) of cefiderocol increased in a dose-proportional manner, and the apparent total body clearance (CL/*F*) was approximately constant. The CL/*F* values of cefiderocol were almost similar to those of cefepime. These PK parameters of cefiderocol were similar between the two infection models. For the PK/PD analysis, the PK parameters of cefiderocol and cefepime in the thigh infection model were estimated using a 2-compartment model with 1st-order absorption and a 1-compartment model with 1st-order absorption, respectively. For cefiderocol, the estimated central volume (*V_c_*), elimination rate constant (*k*_el_), transfer rate between compartments 1 and 2 (*k*_12_), transfer rate between compartments 2 and 1 (*k*_21_), and absorption rate constant (*k_a_*) were 0.569 liter/kg, 1.6 h^−1^, 0.0214 h^−1^, 0.482 h^−1^, and 8.96 h^−1^, respectively. As the PK of cefepime were nonlinear with the dosage, the PK parameters of cefepime were estimated for two dose groups (the 10- and 100-mg/kg-of-body-weight groups or the 1,000-mg/kg group). The PK parameters for the 10- and 100-mg/kg groups were used for the calculation of the PK/PD parameters at doses of up to 240 mg/kg/time. Otherwise, the PK parameters for the 1,000-mg/kg group were used. For the 10- and 100-mg/kg cefepime groups, *V_c_*, *k*_el_, and *k_a_* were 0.430 liter/kg, 2.07 h^−1^, and 7.81 h^−1^, respectively. In 1,000-mg/kg group, *V_c_*, *k*_el_, and *k_a_* were 1.10 liter/kg, 1.05 h^−1^, and 3.19 h^−1^, respectively.

**TABLE 2:** PK parameters of cefiderocol and cefepime following a single subcutaneous administration to neutropenic mice infected with P. aeruginosa SR27016

Compound	Infection model	Dose (mg/kg)	*C*_max_ (μg/ml)	*t*_1/2_^*a*^ (h)	CL/*F* (ml/h/kg)
Cefiderocol	Thigh	4	4.42	0.43	882
	Thigh	40	50.6	0.50	801
	Thigh	400	381	0.56	934
	Lung	4	4.01	1.20	805
	Lung	40	41.7	0.28	670
	Lung	400	310	0.57	921
	Lung	600	386	0.44	972
Cefepime	Thigh	10	14.3	0.45	798
	Thigh	100	145	0.23	853
	Thigh	1,000	808	0.74	1,029

a *t*_1/2_, half-life.

### Protein binding ratio of cefiderocol.

The adsorption ratio of cefiderocol to the ultrafiltration device was 3.9% or below at all concentrations. Therefore, the protein binding ratio of cefiderocol was measurable by the ultrafiltration method. The protein binding ratio of cefiderocol in mice was determined to be 38.7% ± 1.1%, which was similar to that of cefepime (12).

### Determination of the predictive PK/PD parameter for *in vivo* efficacy.

To observe the PK/PD profile of cefiderocol in comparison with that of other β-lactams, dose fractionation studies with cefiderocol and cefepime were performed using the neutropenic murine thigh infection models in which the infections were caused by cefepime-susceptible P. aeruginosa SR27016. Cefepime was selected as a comparator because it has a chemical structure similar to that of cefiderocol. The strain was selected because it has the same susceptibility to cefiderocol in both ID-CAMHB and CAMHB. This could lead to the PK/PD observation without considering the effect of iron on the antibacterial activity.

**FIG 1:**
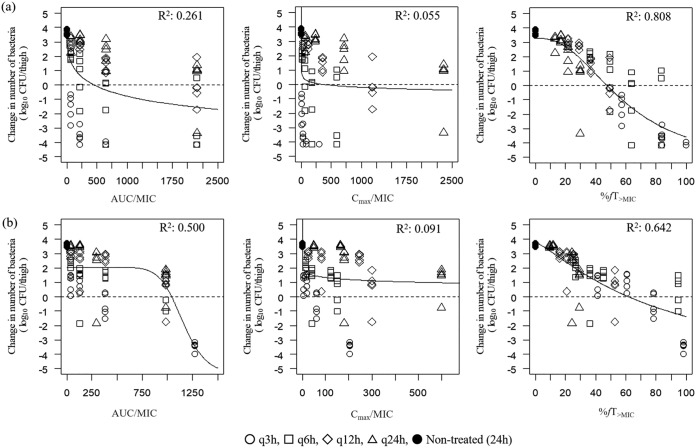
Correlation of PK/PD parameters with efficacy for cefiderocol (a) and cefepime (b) in a neutropenic murine thigh infection model in which infection is caused by Pseudomonas aeruginosa SR27016. Treatment was initiated at 2 h postinfection. Cefiderocol and cefepime were subcutaneously administered once daily (q24h), twice daily (q12h), 4 times a day (q6h), and 8 times a day (q3h). Each point represents data for each mouse. *R*^2^, square of the correlation coefficient.

The relationship between efficacy and three PK/PD indices, the free drug peak level divided by the MIC (*fC*_max_/MIC), the area under the free concentration-time curve over 24 h divided by the MIC (*f*AUC/MIC), and the cumulative percentage of a 24-h period that the free drug concentration in plasma exceeds the MIC (%*fT*_>MIC_), are shown in Fig. 1a for cefiderocol and Fig. 1b for cefepime. The value of the square of the correlation coefficient (*R*^2^) of %*fT*_>MIC_ was the highest for both cefiderocol and cefepime, indicating that %*fT*_>MIC_ is the PK/PD parameter which correlates best with efficacy compared to the other PK/PD parameters tested. The %*fT*_>MIC_ of cefiderocol required for a static effect and a 1-log_10_ reduction was 47.5% and 57.6%, respectively, and that of cefepime was 61.7% and 87.7%, respectively.

### Correlation of *in vivo* efficacy with MIC determined under iron-depleted conditions.

**FIG 2:**
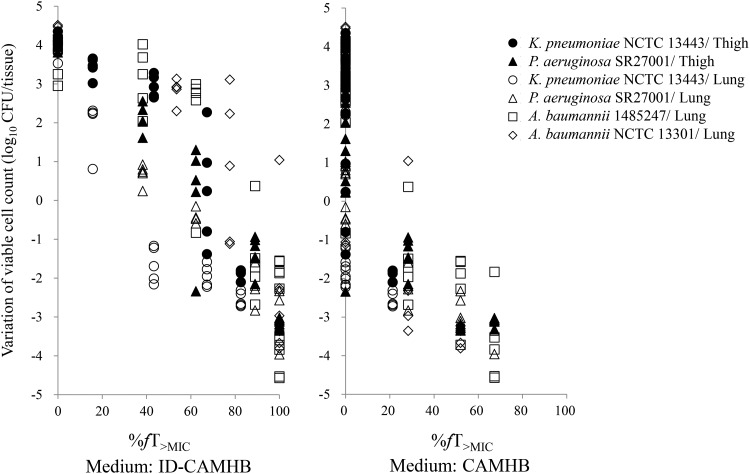
Comparison of the %*fT*_>MIC_ of cefiderocol between MIC values in ID-CAMHB and CAMHB in neutropenic murine thigh and lung infection models.

To determine the appropriate MIC culture condition that has a good correlation with *in vivo* efficacy, the %*fT*_>MIC_ values required for *in vivo* efficacy in both the thigh and lung models were determined using 4 strains (Klebsiella pneumoniae NCTC 13443, P. aeruginosa SR27001, A. baumannii 1485247, and A. baumannii NCTC 13301) which showed 16-fold or higher MIC differences between ID-CAMHB and CAMHB (Fig. 2). When the MIC values determined in ID-CAMHB were used, a %*fT*_>MIC_ of 34.7 to 87.5% was required for a 1-log_10_ reduction. In the case of MICs determined in CAMHB, a %*fT*_>MIC_ of 28.0% or less was required for a 1-log_10_ reduction, and the spread in viable cell counts ranging from a 3-log_10_ reduction to a 5-log_10_ increase was observed at a %*f*T_>MIC_ of 0%.

### Magnitudes of %*fT*_>MIC_ required for efficacy against multiple strains.

**FIG 3:**
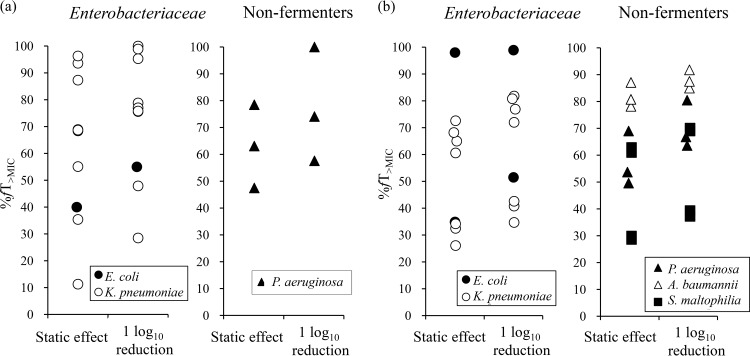
Magnitude of the %*fT*_>MIC_ required for the efficacy of cefiderocol against multiple strains of Enterobacteriaceae and nonfermenters in the neutropenic murine thigh (a) and neutropenic lung infection (b) models.

Additional evaluation of the %*fT*_>MIC_ magnitudes based on ID-CAMHB against the multiple strains listed in Table 1 was conducted using thigh or lung infection models. The %*fT*_>MIC_ of cefiderocol required to achieve a static effect and a 1-log_10_ reduction were determined in the thigh infection models in which the infections were caused by 1 Escherichia coli strain, 9 K. pneumoniae strains, and 3 P. aeruginosa strains (including strain SR27016, used for the dose fractionation study) (Fig. 3a). In order to observe the influence of carbapenem resistance, 6 carbapenem-susceptible strains and 7 CR strains were used. The mean %*fT*_>MIC_ of cefiderocol required for a static effect against the 10 strains of Enterobacteriaceae was 62.5% ± 27.4%, which was similar to that against 3 strains of P. aeruginosa (63.0% ± 15.5%). Similarly, the mean %*fT*_>MIC_ of cefiderocol required for a 1-log_10_ reduction against Enterobacteriaceae (73.3% ± 23.3%) was similar to that required for a 1-log_10_ reduction against P. aeruginosa (72.2% ± 21.4%). Overall, the mean %*fT*_>MIC_ for a 1-log_10_ reduction was higher (*P* = 0.045) for carbapenem-resistant strains (85.2 ± 12.1%) than for carbapenem-susceptible strains (61.3% ± 25.0%).

The evaluation using murine lung infection models was conducted to encompass the bacterial species of A. baumannii and S. maltophilia because thigh infection models with these bacterial species were not able to be established in our experiments. The lung infection models were evaluated with a total of 19 strains (18 CR strains): 2 E. coli, 7 K. pneumoniae, 3 P. aeruginosa, 3 A. baumannii, and 4 S. maltophilia strains (Fig. 3b). In the lung infection model, the mean %*fT*_>MIC_ of cefiderocol required for a 1-log_10_ reduction was 64.4% ± 22.5% against the 9 strains of Enterobacteriaceae and 70.3% ± 9.0% against the 3 strains of P. aeruginosa, values which were not significantly different from those obtained in the thigh infection model (*P* = 0.78). The mean %*fT*_>MIC_ required for a 1-log_10_ reduction against A. baumannii and S. maltophilia were 88.1% ± 3.4% and 53.9% ± 18.1%, respectively.

## DISCUSSION

Based on our results using the conventional dose fractionation methodology in the murine thigh infection model with P. aeruginosa, the %*fT*_>MIC_ was shown to be the PK/PD parameter which best correlated with *in vivo* efficacy for cefiderocol, as well as cefepime (Fig. 1). This could be identical to the previous observation that the efficacy of cefiderocol was enhanced in a rat respiratory tract infection model under the conditions used to cause the prolonged %*fT*_>MIC_ (13).

The MICs determined in ID-CAMHB rather than the MICs determined in CAMHB were more predictive of *in vivo* efficacy, as evidenced in Fig. 2. This was reasonable, as iron starvation in the animals is mimicked by using ID-CAMHB. Actually, the iron concentrations in ID-CAMHB and CAMHB are reported to be approximately 0.02 and 0.15 mg/liter, respectively (14), and it has been reported that the upregulation of iron transporters caused the decrease in the MIC of cefiderocol in ID-CAMHB (5, 15). On the other hand, it has been reported that iron starvation at the infection site in humans is due to the production of the iron-binding proteins, such as transferrin and lactoferrin (16). Cefiderocol was shown to be active against CR Gram-negative isolates even in the presence of apotransferrin to cause iron-depleted conditions as well as in ID-CAMHB (8, 9). ID-CAMHB was used because the MIC in the apotransferrin-containing medium did not show a reproducible MIC against quality control strains (data not shown). From these results, ID-CAMHB was selected as the appropriate medium to evaluate the activity of cefiderocol under iron-depleted conditions.

The present studies demonstrated that the %*fT*_>MIC_ values of cefiderocol required for efficacy did not vary significantly among the Enterobacteriaceae and P. aeruginosa strains, as observed in the murine thigh and lung infection models. This was reasonable because the same free concentrations of β-lactams have been observed in plasma, thigh, and lungs (17). It has also been reported that the free plasma concentrations of imipenem in plasma could be substituted in a conventional PK/PD analysis even in the lung infection model (18). Although A. baumannii and S. maltophilia were evaluated only in lung infection models, the mean %*fT*_>MIC_ values of cefiderocol required for a 1-log_10_ reduction against A. baumannii were higher than those required for a 1-log_10_ reduction against Enterobacteriaceae and P. aeruginosa by 18% to 24%, and those required for a 1-log_10_ reduction against S. maltophilia were lower by 11% to 16%. However, these differences were within the variation between individual strains of Enterobacteriaceae and P. aeruginosa (*P* = 0.15). It is not clear whether these differences are due to the different profiles between bacterial species or not, as the number of the test strains of each bacterial species was not large. To clarify the slightly significant difference between carbapenem-susceptible strains and carbapenem-resistant strains, additional studies using larger number of test strains will be needed. Monogue et al. reported the efficacy of cefiderocol against larger numbers of strains (93 strains, including carbapenem-resistant strains) with variable MICs under human PK exposure conditions in neutropenic murine thigh infection models (19). The results showed that efficacy was observed against strains with MICs of ≤4 μg/ml and that decreased efficacy was observed against strains with MICs of >4 μg/ml, irrespective of the bacterial species, including E. coli, K. pneumoniae, P. aeruginosa, and A. baumannii, or carbapenem susceptibility, suggesting that the similar target %*fT*_>MIC_ values of cefiderocol could be required for efficacy for these bacterial species (19). However, some limitations of this study can be noted. First, the variation of the %*fT*_>MIC_ values seemed to be large between individual strains of each bacterial species. The similar variation was also reported for other β-lactams against Gram-negative bacteria, although the reason has not been clarified (20, 21). Further studies will be necessary to investigate more appropriate %*fT*_>MIC_ values by adding various strains. Second, the evaluation used only mouse lung infection models for A. baumannii and S. maltophilia due to their poorer growth in thigh infection models than in lung infection models. An evaluation using mouse thigh infection models will be needed even for these bacterial species. Third, the inoculum size used in the lung infection models ranged from 10^6^ to 10^7^ CFU/lungs in the previous studies (20–22). However, since 10^5^ to 10^6^ CFU/lungs was used in this study, the possibility of overestimation cannot be excluded. Fourth, information on the concentration at the infection site is not available. To clarify details about the PK/PD profile and differences between the infection sites, additional pharmacokinetic data, such as the concentration in epithelial lining fluid, are needed.

In conclusion, the PK/PD index that best correlated with the *in vivo* efficacy of cefiderocol was the %*fT*_>MIC_, calculated by using the MIC determined under iron-deficient conditions, and the %*fT*_>MIC_ required for efficacy was almost similar among the different bacterial species in the neutropenic thigh and lung infection models. These results suggest that these PK/PD parameters will be useful for the prediction of *in vivo* efficacy from the *in vitro* MIC under iron-depleted conditions. These data support the suggestion that cefiderocol is a promising siderophore cephalosporin for the treatment of Gram-negative bacterial infections, including those caused by multidrug-resistant strains.

## MATERIALS AND METHODS

### Antibiotics.

The following antibiotics were obtained from the indicated suppliers: cefiderocol and [thiazole-^14^C]cefiderocol were from Shionogi & Co., Ltd., meropenem and cefepime pentahydrate for *in vitro* MIC studies were from U.S. Pharmacopeia (Rockville, MD, USA), and cefepime hydrochloride for *in vivo* studies was from Bristol-Myers K.K. (New York, NY, USA). These antibiotics, including cefiderocol, were reconstituted with saline. All concentrations of the antibiotics are expressed in terms of the concentrations of their free forms.

### Microorganisms.

The tested strains were as follows (Table 1): 3 strains of E. coli, including an NDM producer; 10 strains of K. pneumoniae, including NDM and KPC producers; 4 strains of P. aeruginosa, including a metallo-β-lactamase (IMP, VIM) producer; 3 strains of A. baumannii, including OXA-23 and OXA-24 producers; and 4 strains of S. maltophilia. Global pandemic clones, such as E. coli sequence type 14 (ST14) and K. pneumoniae, ST258 were also included (23, 24).

### Animals.

Five-week-old, specific-pathogen-free, male Jcl:ICR mice (weight, 17 to 20 g) were obtained from CLEA Japan, Inc. (Tokyo, Japan). Three to five mice per group were used in all experimental infection models. All studies with animals were approved by the Institutional Animal Care and Use Committee of Shionogi & Co., Ltd.

### Susceptibility testing.

MICs were determined by broth microdilution methods according to CLSI recommendations (25). In the case of cefiderocol, ID-CAMHB was used, in addition to CAMHB, as recommended by CLSI. For comparison of culture conditions, the MICs of cefiderocol were also determined using CAMHB.

### Thigh infection model.

The neutropenic mouse thigh infection model outlined by Andes and Craig was tested (26). Mice were rendered neutropenic by intraperitoneal injection of cyclophosphamide (Shionogi, Osaka, Japan) at 150 and 100 mg/kg at 4 days and 1 day before infection, respectively. The mice were anesthetized by inhalation of isoflurane and then infected by intramuscular injection of 0.1 ml of bacterial suspension into the thigh. The bacterial inoculum was prepared with Mueller-Hinton broth (MHB; Becton, Dickinson and Company, NJ, USA). A total of 12 strains (1 E. coli strain, 9 K. pneumoniae strains, and 2 P. aeruginosa strains) were studied. The challenge doses of these strains were approximately 10^6^ CFU/thigh.

### Lung infection model.

The neutropenic mouse lung infections outlined by Koomanachai et al. (27) and Drusano et al. (28) were tested. Mice were rendered neutropenic as noted above and anesthetized by intramuscular injection of the mixture of tiletamine, zolazepam, and xylazine. The mice were infected by intranasal instillation of 0.07 ml of bacterial suspension. For the infections by E. coli, K. pneumoniae, A. baumannii, and S. maltophilia, the inoculum was prepared with 4.5% porcine gastric mucin (MP Biomedicals, Inc., CA, USA) in order to prevent rapid bacterial clearance by immune cells. For infections by P. aeruginosa, the inoculum was prepared with saline. A total of 19 strains (2 E. coli, 7 K. pneumoniae, 3 P. aeruginosa, 3 A. baumannii, and 4 S. maltophilia strains) were studied. The challenge doses ranged from approximately 10^5^ to 10^6^ CFU/lung.

### Pharmacokinetic study.

The PK parameters of cefiderocol in neutropenic mice infected with P. aeruginosa SR27016 in the thigh or lung were analyzed from the plasma concentrations determined following administration of a single subcutaneous dose. Cefiderocol was administered to thigh-infected mice (4, 40, and 400 mg/kg) and lung-infected mice (4, 40, 400, and 600 mg/kg) 2 h after infection. Cefepime was also studied in the thigh-infected mice at doses of 10, 100, and 1,000 mg/kg. At each sampling time, mice (*n* = 3 per each group) were anesthetized by inhalation of isoflurane. Blood samples were collected at 0.083, 0.25, 0.5, 1, 2, 4, and 6 h after drug administration and centrifuged at 3,500 rpm for 10 min at 4°C to obtain a plasma sample. The plasma concentrations of cefiderocol and cefepime were determined by liquid chromatography-tandem mass spectrometry (LC-MS/MS) (29, 30). Briefly, after the proteins were removed from mouse plasma samples by precipitation using 0.1% trifluoroacetic acid and methanol, the cefiderocol and cefepime concentrations were determined by LC-MS/MS using mobile phase A (water-heptafluorobutyric acid [HFBA]) and mobile phase B (acetonitrile-HFBA). S-649266-*d*_12_ sodium was used as the internal standard for cefiderocol. The lower limits of quantification (LLOQ) of cefiderocol and cefepime were 0.05 μg/ml and 0.5 μg/ml, respectively. The analytical methods with cefiderocol and cefepime were fully validated, and within-run and between-run precision and accuracy were ≤15% (LLOQ, ≤20%) and within 100% ± 15% (LLOQ, within 100% ± 20%), respectively.

### Plasma protein binding.

The protein binding ratio of cefiderocol in mouse plasma was determined by ultrafiltration and a radioactivity measurement method with [thiazole-^14^C]cefiderocol. Briefly, mouse plasma mixed with [thiazole-^14^C]cefiderocol was incubated at 37°C on a water bath for 15 min. The incubated samples (*n* = 3; cefiderocol concentration, 1 μg/ml) were dispensed into ultrafiltration devices (Centrifree YM-30: Merck Millipore, Germany) and centrifuged (1,800 × *g*, 37°C, 15 min). The filtrate was collected, and the radioactivity of the filtrate mixed with Hionic-Fluor scintillator was measured using liquid scintillation counter. The protein binding ratio was calculated from the radioactivity in the test sample before centrifugation and in the filtrate after centrifugation. Nonspecific binding of cefiderocol to the filter device was assessed using a phosphate-buffered saline solution of cefiderocol. For cefepime, the protein binding ratio of 29% that was previously reported was used (12).

### Treatment protocols.

Both murine thigh and lung infection models were used for PK/PD analyses. Subcutaneous administration of antibiotics was initiated at 2 h postinfection. For the dose fractionation study, mice were treated for 24 h with cefiderocol or cefepime, administered as multiple-dosing regimens. The total daily doses of cefiderocol and cefepime were 24, 80, 240, and 800 mg/kg and 48, 160, 480, and 1,600 mg/kg, respectively. Each dose was given once daily (q24h), as two equally divided doses every 12 h (q12h), as four equally divided doses every 6 h (q6h), and as eight equally divided doses every 3 h (q3h). To determine the magnitude of the cumulative percentage of a 24-h period that the free drug concentration in plasma exceeds the MIC (%*fT*_>MIC_) required for efficacy, cefiderocol was administered as multiple doses of 0.1 to 600 mg/kg every 3 h. Control mice were treated with 0.9% saline. At 24 h after the initial treatment, mice (*n* = 5 per each group) were euthanized and the numbers of viable bacteria in tissue samples were counted.

### Data analysis.

PK parameters (*C*_max_, CL/*F*) were calculated by the use of WinNonlin software (Pharsight, NJ, USA), based on a noncompartmental model with uniform weighting. For the PK/PD analysis, the PK parameters estimated from the free plasma concentrations determined in the thigh infection model were used. The sigmoid maximum effect (*E*_max_) model was fit to the exposure and pharmacodynamic responses to determine the %*fT*_>MIC_ values of cefiderocol and cefepime resulting in a static effect or a 1-log_10_ reduction. A one-way analysis of variance (ANOVA) was used to compare the data among groups (Enterobacteriaceae, P. aeruginosa, A. baumannii, and S. maltophilia). *P* values of *<*0.05 were considered statistically significant.
